# Establishment and Application of an Indirect ELISA for the Detection of Antibodies to Porcine *Streptococcus suis* Based on a Recombinant GMD Protein

**DOI:** 10.3390/ani13040719

**Published:** 2023-02-17

**Authors:** Nihua Dong, Zhaofei Wang, Qing Sun, Xiaojun Chen, Hailong Zhang, Jiayang Zheng, Xinya Zhang, Yafeng Qiu, Zongjie Li, Beibei Li, Ke Liu, Donghua Shao, Jianchao Wei, Jianhe Sun, Zhiyong Ma

**Affiliations:** 1 Shanghai Veterinary Research Institute, Chinese Academy of Agricultural Science, Shanghai 200241, China; 2 Shanghai Key Laboratory of Veterinary Biotechnology, School of Agriculture and Biology, Shanghai Jiao Tong University, Shanghai 200240, China

**Keywords:** *Streptococcus suis*, prokaryotic expression, indirect ELISA, serological survey

## Abstract

**Simple Summary:**

This study developed an indirect ELISA (GMD-ELISA) to detect the *Streptococcus suis* (*S. suis*) antibody. The antigen—antibody response was optimized using checkerboard titration. This method has strong specificity and can detect the main epidemic strains of *S. suis* in China—type 2, type 7, and type 9—compared with using the commercial *Streptococcus suis* ELISA type 2 kit. The GMD-ELISA method has high sensitivity and good repeatability. The novel GMD-ELISA method provides technical support for rapid diagnosis and epidemiological investigation.

**Abstract:**

*S. suis* is an important zoonotic pathogen from sick and recessive carrier pigs that poses a serious threat to animal husbandry production and public health. It usually causes horizontal transmission among pigs. The morbidity and mortality of this disease are very high. Human infection is caused through direct or indirect contact with sick pigs. The two large-scale outbreaks in China were due to the outbreak of *S. suis* on pig farms, which spread to human infection; thus, detecting *S. suis* in pig herds is crucial. At present, the commercial *S. suis* ELISA type 2 kits on the market can only detect single serotypes, high probabilities of interaction reactions, and biosafety risks when using inactivated *S. suis* as an antigen. Phosphate-3-glyceraldehyde dehydrogenase (GAPDH), muramidase-released protein (MRP), and dihydrolipoamide dehydrogenase (DLDH) are important *S. suis* type 2, *S. suis* type 7, and *S. suis* type 9 protective antigens. This study purified the GMD protein (B-cell-dominant epitopes of GAPDH, MRP, and DLDH antigens) and used a diverse combination of dominant epitopes of the multiple different antigens as coated antigens, improving the sensitivity and safety of the indirect ELISA experiments. An indirect ELISA method (GMD-ELISA) was developed for detecting *S. suis* antibodies. The antigen—antibody response was optimized using checkerboard titration. The results of testing using ELISA for *Salmonella enterica* (*S. enterica*), *Escherichia coli* (*E. coli*), *Staphylococcus aureus* (*SA*), and *Streptococcus pyogenes* (*S. pyogenes*) were all negative, indicating that this method had strong specificity. The results were still positive when the dilution ratio of *S. suis*-positive serum reached 1:6, 400, thus indicating that the method had high sensitivity. The results of the reproducibility assay for indirect ELISA showed that the intra-assay coefficient of variation and the inter-assay coefficient of variation were less than 10%, indicating that the method had good repeatability. We investigated the seroprevalence of *S. suis* in 167 serum samples collected in East China, and 33.5% of the samples were positive for antibodies against *S. suis*, indicating that the prevalence of *S. suis* is high in pig farms in Eastern China. The novel GMD-ELISA is a convenient, sensitive, and specific diagnostic method that provides technical support for rapid diagnosis and epidemiological investigation.

## 1. Introduction

*S. suis* is an encapsulated Gram-positive bacterium. According to the different capsular antigens, *S. suis* can be divided into 35 serotypes, of which serotypes 2, 7, and 9 are the main pathogenic bacterial groups in pigs [1,2,3]. It can cause disease in animals and humans and can be transmitted to humans through wounds and the digestive tract, causing clinical symptoms such as sepsis, pneumonia, endocarditis, arthritis, meningitis, and death [4,5,6,7]. Prior to 2005, over 200 cases were reported in European and Asian countries. Two large-scale outbreaks of human *S. suis* infection occurred in China in 1998 and July 2005, resulting in widespread fatalities [8,9,10]. Repeated outbreaks have raised worldwide concern about its potential as an emerging zoonotic pathogen.

Accurately and rapidly detecting *S. suis* is very important in the early diagnosis and treatment of infection. Traditional microbiological and biochemical analyses are laborious, time-consuming, and have low specificity or sensitivity [11]. Ju et al. (2010) developed a colloidal gold immunochromatography method for rapidly detecting *S. suis* type 2, but the sensitivity of this method was poor and it can only detect a positive result when the bacterial concentration is greater than 10^5^ cfu/mL [12]. Therefore, developing a rapid detection method for *S. suis* for real-time studies and serological monitoring is necessary.

Over the past few decades, immunoassays have become routine methods for detecting pathogens. Indirect ELISA is a quick and simple method for detecting antibodies or antigens while screening many samples in a single experiment [13,14]. It can be used for rapidly evaluating a vaccine’s immune effect by detecting the antibody level and serum titer changes caused by vaccine immunization.

The enzyme phosphate-3-glyceraldehyde dehydrogenase (GAPDH) plays a significant role in the glycolytic pathway and is also a surface-related factor in the interaction of pathogenic *Streptococci* with host proteins and cells [15]. Muramidase-released protein (MRP) is an important virulence factor of *S. suis* and both GAPDH and MRP have been proven to be related to adhesion [16]. Moreover, FAD-dependent enzyme dihydrolipoamide dehydrogenase (DLDH) is one of the components of the pyruvate dehydrogenase system, which participates in cellular respiratory energy metabolism. In *Neisseria pneumoniae* (*N.pneumoniae*), DLDH is speculated to transport sugars and has been shown to have high immunogenicity [17].

Wang et al. found that the GMD protein showed effective immune protection against *S. suis* type 2, *S. suis* type 7, and *S. suis* type 9 in zebrafish. Immunizing mice and pigs with monophosphoryl lipid A (MPLA) as an adjuvant of the TLR4 agonist induced a strong innate immune response and had a good protective effect for both mice and pigs [18]. In this paper, the GMD protein was purified, with the aim of developing an indirect ELISA test as a reliable technical method for serologically investigating the *S. suis* disease.

## 2. Materials and Methods

### 2.1. Serum Samples and Materials

*S. suis* type 2 (*ZY05719 strain*), *S. suis* type 7 (*SH04815 strains*), and *S. suis* type 9 (*SH26 strain*) and the corresponding reference antisera of serotypes 2, 7, and 9 (SS2, SS7, SS9) were obtained by Professor Sun Jianhe from Shanghai Jiao Tong University.

To establish the GMD-ELISA protocol, 30 antisera of *S. suis* serotypes 2, 7, and 9 (SS2-P1~SS2-P10, SS7-P1~SS7-P10, SS9-P1~SS9-P10) from naturally infected pigs and 34 negative sera (SS-N1~SS-N34), which were identified using the agglutination test described previously [19], were used (Supplementary Table S1). The positive sera for *S. enterica*, *E. coli*, *SA*, and *S. pyogenes* were provided by the laboratory of the Shanghai Veterinary Research Institute. In the conformance rate experiment, 40 serum samples were collected from farms with disease outbreaks and 60 serum samples were collected from farms without disease outbreaks. One hundred and sixty-seven samples of pig serum from (Shandong, Hebei, Zhejiang, Shanghai, Jiangsu, Guangdong) East China were stored by the Shanghai Veterinary Research Institute. 

### 2.2. Expression and Purification of Recombinant GMD Protein

The recombinant plasmid containing the GMD gene was constructed by Wang Zhaofei of Shanghai Jiao Tong University. TMHMM, SignalP, ABCpred, Beppred 2.0, Sopma, and DNASTAR were used to predict the B-cell epitopes of GAPDH MRP and DLDH antigens; finally, 15 B-cell antigen-dominant epitopes were obtained. According to the predicted dominant B-cell epitopes of GAPDH, MRP, and DLDH proteins, all the epitopes were combined and spliced in sequence in the order of GAPDH–MRP–DLD and the epitopes were connected in a series using GGGG flexible fragments to construct the recombinant plasmid GMD-pET-28a [18]. The recombinant plasmid was transferred to *E. coli* BL21 to induce GMD protein expression. In this study, the GMD proteins were purified on a nickel column using a His-Bind Purification Kit (Bio-Rad, Berkeley, CA, USA) and confirmed using sodium dodecyl sulfate polyacrylamide gel electrophoresis (SDS-PAGE).

### 2.3. Optimizing ELISA Indirect Conditions

After optimization, the ELISA assay was performed in 96-well microtiter plates according to the method described in a previous study [14]. In order to establish the iELISA, checkerboard titration was performed to determine the optimal concentrations of the coating antigen, sera, and secondary antibodies. The recombinant GMD protein was serially diluted in a twofold series to 6.72, 3.36, 1.68, 0.84, 0.42, and 0.21 μg/mL. The standard positive serum for *S. suis* types 2 (SS2), *S. suis* 7 (SS7), and *S. suis* 9 (SS9) and the negative serum for *S. suis* were also diluted from 1:50 to 1:6400 in the same manner. The secondary antibody horseradish peroxidase (HRP)-conjugated goat anti-swine IgG antibody (Sigma-Aldrich, St. Louis, MO, USA) was diluted to 1:5000, 1:10,000, 1:15,000, and 1:20,000. Briefly, the 96-well microtiter plates were coated with 100 μL of purified GMD protein at 4 °C overnight. The wells were washed three times with phosphate-buffered saline containing 0.05% Tween 20 (PBST) and blocked with 5% skimmed milk for 2 h at 37 °C. 

Next, the wells were washed three times and incubated with 100 μL of diluted sera at 37 °C for 1 h. The wells were then washed and incubated with 100 μL of diluted secondary antibody at 37 °C for 1 h. The wells were again washed three times using PBST and 200 μL of 3,3′,5,5′-tetramethylbenzidine (TMB) (KPL, Gaithersburg, MD, USA) reaction solution was added. After 15 min of incubation in the dark at room temperature, the reactions were stopped by adding 50 μL of 2 M H2SO4. Then, the OD value was measured at 450 nm using a spectrophotometer. The optimal concentrations of the coating antigen, sera, and secondary antibody were selected as those that provided the highest ratio of OD450 values between the positive and the negative controls (P/N). 

### 2.4. Determining the Critical Value

The optimized GMD-ELISA method was used to detect 34 *S. suis*-negative sera with a clear background, and the mean OD450 nm value (X) and standard deviation (SD) were calculated. When the mean OD450 nm value of the to-be-evaluated serum equaled or exceeded? $\bar{X}$ + 3SD, it was considered positive. When the mean OD450 nm value of the serum to be evaluated was less than? $\bar{X}$ + 3SD, it was considered negative.

### 2.5. Specificity Analysis 

A total of 35 specific serum samples (the negative serum for *S. suis*, *S. enterica*, *E. coli*, *SA*, and *S. pyogenes*, Supplementary Table S1) were used to evaluate the specificity of the ELISA. 

### 2.6. Sensitivity Analysis

The positive sera of *S. suis* serotypes 2, 7, and 9 (SS2-P1~SS2-P3, SS7-P1~SS7-P3, SS9-P1~SS9-P3) were diluted at 1:100, 1:200, 1:400, 1:800, 1:1600, 1:3200, 1:6400, 1:12,800, 1:25,600, and 1:51,200 and detected using the established GMD-ELISA method to determine the minimum detection amount. The sensitivity of the commercially available *S. suis* type 2 ELISA kit was also analyzed.

### 2.7. Reproducibility Assay for Indirect ELISA 

The GMD-ELISA’s reproducibility was evaluated using six serum samples, which were chosen from the present primary tests. The coefficient of variation (CV) was used to evaluate the intra- and inter-assay variation. Each sample was evaluated on each of the three plates on separate occasions to determine the inter-assay CV, while three replicates within the same plate were used to calculate the intra-assay CV. The mean sample/positive control (S/P) ratios and standard deviations (SD) were also calculated.

### 2.8. Conformance Rate Experiment 

To establish this experiment, antibodies from 100 clinical pig serum samples were simultaneously detected by using the commercial *S. suis* type 2 ELISA kit and the GMD-ELISA method. The *S. suis*-positive and -negative sera were used as positive and negative controls with two replicates.

### 2.9. Elimination of GMD Antibodies after S. suis Type 2 Challenge in Clinically Healthy Pigs

Three clinically healthy sows were selected and numbered as one, two, and three, and each was intravenously injected with the 5 × 10^6^ CFU strain of *S. suis* type 2 ZY05719. They were each tracked and monitored for the extinction of the serum GMD antibodies.

### 2.10. Statistical Analysis 

All the results are presented as the means ± standard errors of the means (SE) of triplicate experiments. The data were analyzed using GraphPad Prism (GraphPad Software 8.0.1, San Diego, CA, USA) and SPSS 22.0 (IBM Corp., Armonk, NY, USA). The statistical significance was evaluated using a one-way ANOVA as an analysis of variance.

## 3. Results

### 3.1. Expression and Purification of the Recombinant GMD Protein

**Figure 1:**
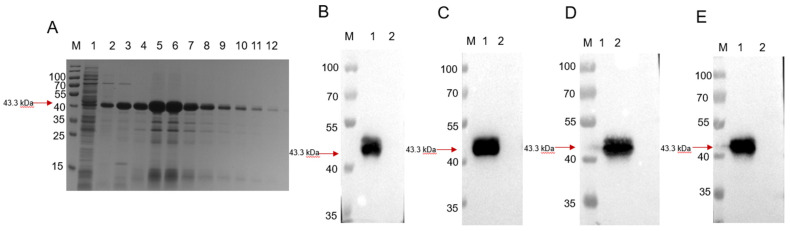
Purification and validation of the recombinant GMD protein. Electrophoretic diagram of GMD protein purified using Ni column SDS-PAGE (**A**) and identified using Western blot with SS2 (**B**), SS7 (**C**), SS9 (**D**), and anti-His antibody (**E**). (**A**) M: 180 kDa protein marker; 1: flowing liquid; 2:20 mM imidazole eluant; 3:40 mM imidazole eluant; 4:60 mM imidazole eluant; 5–12: 200 mM imidazole eluant. (**B**) M: 180 kDa protein marker; 1: Purified GMD protein; 2: uninduced GMD protein. (**C**) M: 180 kDa protein marker; 1: Purified GMD protein; 2: uninduced GMD protein. (**D**) M: 180 kDa protein marker; 1: Purified GMD protein; 2: uninduced GMD protein. (**E**) M: 180 kDa protein marker; 1: Purified GMD protein; 2: uninduced GMD protein.

The GMD protein was purified by elution using an imidazole gradient. The SDS-PAGE analysis showed that the recombinant GMD protein had an approximate molecular mass of 43.3 kDa, as shown in Figure 1A, from lanes 2 to 11. The Western blot analysis using the *S. suis* type 2 positive serum (SS2), *S. suis* type 7 positive serum (SS7), *S. suis* type 9 positive serum (SS9), and anti-His antibody revealed that the shifted band is the recombinant GMD protein, as seen in Figure 1A–E.

### 3.2. ELISA Optimization Using Recombinant GMD Protein

**Figure 2:**
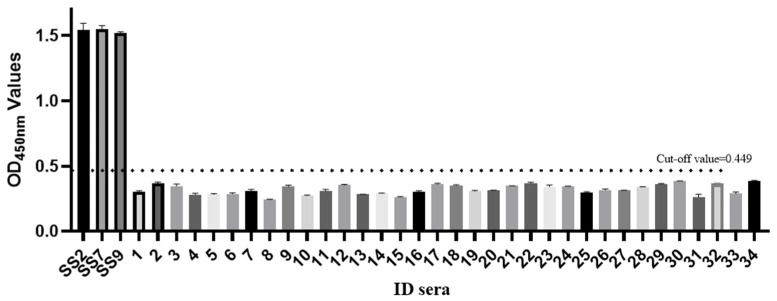
Determination of cut-off value for GMD-ELISA. Cut-off values were evaluated by testing 34 negative control sera. Each value represented the mean absorbance at 450 nm obtained from three replicates of each evaluated serum sample. The cut-off value was defined as mean OD value of all tested negative control sera, plus three standard deviations. The cut-off value (0.449) is represented by dashed lines. Error bars indicate mean and SD.

The optimal working concentration of the antigen coating was shown to be 0.84 μg/mL and the appropriate dilutions of the sera were confirmed to be 1:100, which were determined using checkerboard assays with serial dilutions of antigens and sera. The best dilution of the secondary antibody was 1:5000. Using both *Streptococcus suis*-positive sera and negative sera in the ELISA assay, in the 4 °C condition for 12 h for the positive serum and in the negative serum antigen coating with the maximum P/N value, the GMD protein had the best antigen coating effect working conditions, including for the antigen coating under different optimized temperatures and time conditions. The cut-off value was determined by the sum of the mean OD450 nm value (X) and three standard deviations (SD) of the 34 negative sera. All 34 negative serum samples were further verified using the commercial *S. suis* type 2 ELISA kit and confirmed to be negative for anti-*S. suis* type 2 antibodies. These serum samples were therefore used to determine the cut-off value in Figure 2; they had an average absorbance of 0.323 and a standard deviation of 0.042. The ELISA threshold was 0.323 + 3 × 0.042 = 0.449. All the results were determined based on the principle that the OD450 ratio of the positive to the negative samples should be above 2.1. 

### 3.3. Specificity Analysis of Indirect ELISA

**Figure 3:**
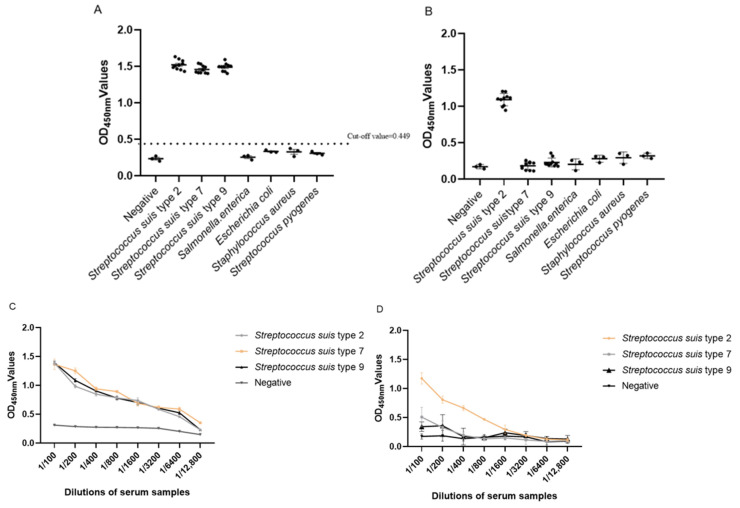
(**A**) GMD-ELISA specificity analysis. The ELISA has cross-reactivity with serum of *S. suis* type 2, type 7, and type 9 and no reactivity with positive sera of other swine viruses, including *S. enterica*, *E. coli*, *SA*, and *S. pyogenes*. (**B**) Commercial *S. suis* type 2 ELISA kit specificity analysis. The ELISA has cross-reactivity with serum of *S. suis* type 2 and no reactivity with positive sera of other swine viruses, including *S. enterica*, *E. coli*, *SA*, and *S. pyogenes*. (**C**) The samples of *S. suis* type 2, *S. suis* type 7, and *S. suis* type 9 were serially diluted twofold from 1:100 to 1:12,800 and detected using the GMD-ELISA method. (**D**) The samples of *S. suis* type 2, *S. suis* type 7, and *S. suis* type 9 were serially diluted twofold from 1:100 to 1:12,800 and detected using the commercial *S. suis* type 2 ELISA kit.

The *S. suis*-positive sera (SS2-P1~SS2-P10, SS7-P1~SS7-P10, SS9-P1~SS9-P10), negative sera, and *S. enterica*, *E. coli*, *SA*, and *S. pyogenes*-positive sera were detected using the established GMD-ELISA method and the results showed good specificity, as seen in Figure 3A. The specificity of the commercial *S. suis* type 2 ELISA kit is shown in Figure 3B. The GMD-ELISA can detect *S. suis* types 2, 7, and 9, while the commercial *S. suis* type 2 kit can only detect *S. suis* type 2 (Figure 3C,D).

### 3.4. Sensitivity Analysis Test of Indirect ELISA

**Figure 4:**
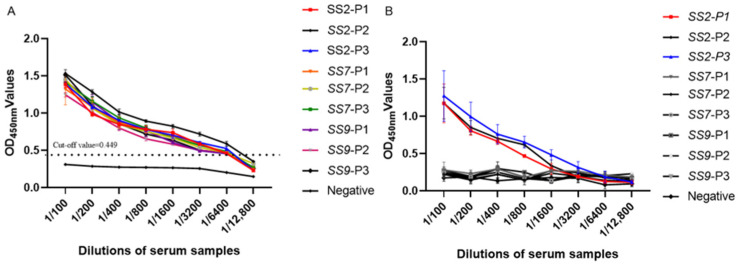
(**A**) Sensitivity analysis. *S. suis*-positive serum was serially diluted twofold from 1:100 to 1:12,800 and detected using the GMD-ELISA method. (**B**) Sensitivity analysis. *S. suis*-positive serum was serially diluted twofold from 1:100 to 1:12,800 and detected using the *S. suis* type 2 ELISA kit.

Different dilutions of *S. suis*-positive serum were evaluated. The detection results showed that the minimum detection amount of this method was 1:6400, as in Figure 4A. The sensitivity of the commercial *S. suis* type 2 ELISA kit was 1:3200, as in Figure 4B. The GMD-ELISA method showed higher sensitivity than the *S. suis* type 2 ELISA kit.

### 3.5. Reproducibility Assay for Indirect ELISA

Three *S. suis*-positive sera and three *S. suis*-negative sera with different antibody levels were used for intra-batch and inter-batch repeatability tests. The calculated results showed that the intra-assay coefficient of variation ranged from 2.42 to 7.07% and the inter-assay coefficient of variation ranged from 3.23 to 6.75%. All were less than 10%, as shown in Table 1, indicating that the method had good repeatability, high accuracy, and could be used for routine detection.

### 3.6. Results of the Conformance Rate Experiments

One hundred clinical swine serum samples were simultaneously detected using a commercial *S. suis* type 2 ELISA kit (Wuhan Keqian Biology, Wuhan, China). The results showed that the *S. suis*-positive sera were all positive, while the *S. suis*-negative sera and blank controls were all negative. The commercial *S. suis* ELISA kit showed that 29 positive sera were detected from 100 clinical serum samples, while 34 positive serum samples were detected using GMD-ELISA(GMD-ELISA was developed for detecting *S. suis* antibodies), as shown in Table 2; the total compliance rate was 95%. 

### 3.7. Eliminating GMD Antibody in Clinical Healthy Pigs Infected with S. suis Type 2

**Figure 5:**
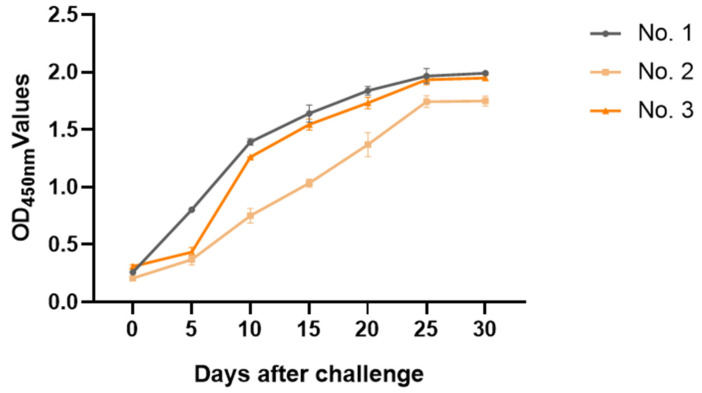
Kinetics of GMD antibody in serum of challenged pigs with *S. suis* type 2.

Three clinically healthy pigs were infected with a 5 × 10^6^ CFU dose of *S. suis* type 2 strain ZY05719. All three pigs showed clinical symptoms such as claudication, happy lying, and lethargy and the symptoms were gradually relieved after seven days. As can be seen from Figure 5, on the 25th day after infection, the serum GMD antibody levels of all the pigs peaked and there were individual differences between them.

### 3.8. Seroprevalence of S. suis in East China

The GMD-ELISA method was used to detect 167 clinical pig serum samples from pig farms in East China, which were kept by the Animal Health Testing Center of Shanghai Veterinary Research Institute, with an *S. suis*-positive rate of 33.5%, as seen in Table 3.

The samples were collected from farms with known or unknown *S. suis* exposure. The GMD-ELISA evaluated 167 pig serum samples.

## 4. Discussion

The diseases caused by *S. suis* have wide transmission channels, with many inducing factors such as high morbidity and mortality that affect the pig industry’s sustainable development [20,21,22,23]. Antibiotics are the main tool to prevent and treat *S. suis* infections, but, with the emergence of drug-resistant strains, many have become ineffective or been banned, so the risk of antibiotic treatment is increased and *S. suis* infection prevention is a major challenge [24,25,26,27].

There are many serotypes of *S. suis* disease. Wei et al. found that the epidemic strains were not fixed but dynamic [28]. An important virulence factor of *S. suis* is MRP, which has good immunogenicity and can be used as a candidate protein of the *S. suis* subunit vaccine [29]. In addition, the MRP and GAPDH genes of different isolates were highly conserved [30]. The DLDH gene supposedly has a sugar transport function and high immunogenicity [31,32]. Good conservation of GAPDH, MRP, and DLDH is also important as they are protective antigens. The current diagnostic methods for *S. suis* infection include routine biochemical analysis, flight mass spectrometry, and polymerase chain reaction [33,34]. These methods are time-consuming, cumbersome, and require the use of sophisticated experimental equipment. Indirect ELISA was used to detect *S. suis* infection, but the specificity of whole bacterial antigens was poor, so this study established a GMD-ELISA based on the recombinant GMD protein, which could quickly detect *S. suis* with good specificity and sensitivity. This was confirmed in a reproducibility test showing that all cross-validation (CV) values for intra-assay and inter-assay were less than 10%. In addition, there was no cross-reactivity with antisera of *S. enterica*, *E. coli*, *SA*, and *S. pyogenes.* The GMD-ELISA method has high sensitivity compared to the commercial *S. suis* ELISA kit. The former can detect *S. suis* type 2, *S. suis* type 7, and *S. suis* type 9.

The inconsistency between the two tests’ results was because the antigens used in the commercial *S. suis* type 2 ELISA kit were inactivated Streptococci and some of the bacterial surface protein structures were destroyed during the inactivation. However, the serotype of the *S. suis* is complex, with more strains of *S. suis* emerging in recent years; the sensitivity of this method is not strong due to the single coating of the bacterial antigen. During the experiment, we selected a reference serum with good characteristics, but the other serotypes contaminating it was inevitable due to the large number of *S. suis* serotypes. This situation leads to differences between the two approaches. This aspect must be continuously improved.

In the elimination experiment of the GMD antibody of *S. suis* type 2 in clinical healthy pigs, the *S. suis* positivity could be clearly distinguished seven days after the initial infection; thus, it can achieve early diagnosis and prevention. At the same time, the *S. suis* positivity cannot be clearly judged in the first 5 days, so this detection method also has certain limitations.

Kataoka Y. used *S. suis* type 2 strain (NCTC 10234) as an antigen to establish an indirect ELISA method [35]; in the antibody elimination experiment, the titer of the ELISA slowly increased within 20 days and then significantly increased after 20 days. However, the ELISA titer of the GMD-ELISA increased significantly and maintained an elevated level after 10 days of challenge. The results showed that the GMD-ELISA method had high sensitivity.

The diseases caused by *S. suis* have caused great harm to the breeding industry and, as an emerging human pathogen, it poses a threat to public health. As a zoonotic disease, the early diagnosis and effective prevention of *S. suis* diseases would maintain public health security by reducing the transmission of large-scale diseases, the economic losses of pig farms, and, most importantly, the probability of human infection.

There are more than 30 serotypes of *S. suis* that cause *S. suis* diseases. Although commercial vaccines are widely used in China and some European countries, disease prevention and control still require development. Since the first human case of *S. suis* was reported in Denmark in 1968, more than 1000 cases have been reported in more than 30 countries with intensive pig production, especially in Southeast Asia [36]. In 2010, there was a serious outbreak of *S. suis* in Northern Thailand, involving 171 human cases [37]. In 2015, more than 500 cases were reported in research facilities in some national and provincial hospitals in Vietnam and 151 patients were found to be affected by *S. suis* [38]. In 1998, there was an outbreak of *S. suis* in Jiangsu Province, China, in which 25 people were infected, 14 died, and approximately 98,000 pigs were infected [35]. Studies of the prevalence of *S. suis* on pig farms in the three main European pig-producing countries—Germany, the Netherlands, and Spain—showed that 3.3 to 4.0% of pigs in infected farms had *S. suis*-related disease, with mortality rates of 0.5 to 0.9% [39]. In Spain, researchers found that 55.6% of patients worked in pig farms [40].

## 5. Conclusions

In this study, the GMD-ELISA test was used to analyze 167 sera from six provinces in East China from 2018 to 2021 and, of them, 34.1% were positive for anti-*S. suis* IgG antibodies. In East China, *S. suis* mostly occurs in summer and autumn, when the climate is hot and humid, which is conducive to bacterial growth.

These data indicated that the prevalence of *S. suis* was high in East China, which may be due to the climate of this region. The GMD-ELISA method established in this study can be used to rapidly detect and epidemiologically investigate *S. suis* disease in pigs.

## Figures and Tables

**Table 1:** The results of repeating test.

Sample Number	Inter-Assay CV (%)		Intra-Assay CV (%)	
	X ± SD	CV (%)	X ± SD	CV (%)
1	1.150 ± 0.040	3.48%	1.154 ± 0.052	4.51%
2	1.302 ± 0.042	3.23%	1.364 ± 0.033	2.42%
3	1.336 ± 0.037	2.77%	1.359 ± 0.045	3.31%
4	0.285 ± 0.016	5.61%	0.297 ± 0.020	6.73%
5	0.348 ± 0.023	6.61%	0.382 ± 0.027	7.07%
6	0.415 ± 0.028	6.75%	0.432 ± 0.026	6.02%

**Table 2:** The results of the conformance test.

		Commercial *Streptococcus suis* Type 2 ELISA Kit		
		Positive	Negative	Total
**GMD-ELISA**	Positive	29	5	34
	Negative	0	66	66
	Total	29	71	100
	Positive coincidence rate		93%	
	Negative coincidence rate		100%	
	The total coincidence rate		95%	

**Table 3:** Epidemiological investigation of *S. suis* in pig farms in Eastern China, 2018–2021.

Sample Type	No. Samples	No. Positive	Positive Rate
Total number of samples	167	56	33.5%
Vaccinated pigs	42	35	83.3%
Unvaccinated pigs	31	6	19.4%
Unknow	94	15	16%

## Data Availability

All data generated during this study are publicly available. However, the raw data are available from the corresponding author upon reasonable request.

## Supplementary Materials

The following supporting information can be downloaded at: https://www.mdpi.com/article/10.3390/ani13040719/s1; Table S1: Identification of negative and positive serum for Streptococcus suis types 2, 7 and 9.
