# Supplementary material for: Establishment and Application of an Indirect ELISA for the Detection of Antibodies to Porcine Streptococcus suis Based on a Recombinant GMD Protein
Source: Animals (Basel). 2023 Feb 17;13(4):719. doi: 10.3390/ani13040719 (PMC9952749; doi:10.3390/ani13040719)
Supplement: Supplementary file 1 [file animals-13-00719-s001.zip › animals-2064006-supplementary.pdf]

# Supplementary material

Table S1 Identification of negative and positive serum for *Streptococcus suis* types 2, 7 and 9

| Sample | Agglutination test for *S.Suis 2*[#] | Agglutination test for *S.Suis* 7[#] | Agglutination test for *S.Suis* 9[#] | GMD-ELISA |
|---|---|---|---|---|
| SS2* | + | - | - | 1.576 |
| SS2-P1 | + | - | - | 1.466 |
| SS2-P2 | + | - | - | 1.633 |
| SS2-P3 | + | - | - | 1.576 |
| SS2-P4 | + | - | - | 1.506 |
| SS2-P5 | + | - | - | 1.603 |
| SS2-P6 | + | - | - | 1.486 |
| SS2-P7 | + | - | - | 1.516 |
| SS2-P8 | + | - | - | 1.453 |
| SS2-P9 | + | - | - | 1.431 |
| SS2-P10 | + | - | - | 1.536 |
| SS7* | - | + | - | 1.520 |
| SS7-P1 | - | + | - | 1.532 |
| SS7-P2 | - | + | - | 1.543 |
| SS7-P3 | - | + | - | 1.489 |
| SS7-P4 | - | + | - | 1.412 |
| SS7-P5 | - | + | - | 1.423 |
| SS7-P6 | - | + | - | 1.449 |
| SS7-P7 | - | + | - | 1.402 |
| SS7-P8 | - | + | - | 1.432 |
| SS7-P9 | - | + | - | 1.409 |
| SS7-P10 | - | + | - | 1.502 |
| SS9* | - | - | + | 1.521 |
| SS9-P1 | - | - | + | 1.531 |
| SS9-P2 | - | - | + | 1.496 |
| SS9-P3 | - | - | + | 1.593 |
| SS9-P4 | - | - | + | 1.403 |
| SS9-P5 | - | - | + | 1.429 |
| SS9-P6 | - | - | + | 1.433 |
| SS9-P7 | - | - | + | 1.494 |
| SS9-P8 | - | - | + | 1.496 |
| SS9-P9 | - | - | + | 1.503 |
| SS9-P10 | - | - | + | 1.512 |
| SS-N1 | - | - | - | 0.317 |
| SS-N2 | - | - | - | 0.355 |
| SS-N3 | - | - | - | 0.344 |
| SS-N4 | - | - | - | 0.301 |
| SS-N5 | - | - | - | 0.283 |
| SS-N6 | - | - | - | 0.291 |
| SS-N7 | - | - | - | 0.296 |
| SS-N8 | - | - | - | 0.243 |
| SS-N9 | - | - | - | 0.352 |
| SS-N10 | - | - | - | 0.275 |
| SS-N11 | - | - | - | 0.306 |
| SS-N12 | - | - | - | 0.357 |
| SS-N13 | - | - | - | 0.285 |
| SS-N14 | - | - | - | 0.291 |
| SS-N15 | - | - | - | 0.315 |
| SS-N16 | - | - | - | 0.361 |
| SS-N17 | - | - | - | 0.351 |
| SS-N18 | - | - | - | 0.322 |
| SS-N19 | - | - | - | 0.317 |
| SS-N20 | - | - | - | 0.348 |
| SS-N21 | - | - | - | 0.373 |
| SS-N22 | - | - | - | 0.366 |
| SS-N23 | - | - | - | 0.347 |
| SS-N24 | - | - | - | 0.307 |
| SS-N25 | - | - | - | 0.331 |
| SS-N26 | - | - | - | 0.316 |
| SS-N27 | - | - | - | 0.343 |

| | | | | |
|---|---|---|---|---|
| SS-N28 | - | - | - | 0.366 |
| SS-N29 | - | - | - | 0.381 |
| SS-N30 | - | - | - | 0.305 |
| SS-N31 | - | - | - | 0.368 |
| SS-N32 | - | - | - | 0.317 |
| SS-N33 | - | - | - | 0.383 |
| SS-N34 | - | - | - | 0.381 |

“+”, positive; “-”, negative.

“*” means that sera is donated by Professor Sun Jianhe from Shanghai Jiao Tong University.

“#”Antisera from naturally infected pigs,and 34 negative sera were identificed serotypes with reference *S.suis* type 2(ZY05719 strain), *S. suis* type 7 (SH04815 strains) and *S. suis* type 9 (SH26 strain) by agglutination test, as described previously [19]

ELISA: “X” >0.449, positive; “X” <0.449, negative.
